# Live Imaging of Mouse Endogenous Neural Progenitors Migrating in Response to an Induced Tumor

**DOI:** 10.1371/journal.pone.0044466

**Published:** 2012-09-05

**Authors:** Gema Elvira, Isabel García, Marina Benito, Juan Gallo, Manuel Desco, Soledad Penadés, Jose A. Garcia-Sanz, Augusto Silva

**Affiliations:** 1 Department of Cellular and Molecular Medicine, Centro de Investigaciones Biológicas (CIB-CSIC), Madrid, Spain; 2 Laboratory of Glyconanotechnology, CICbiomaGUNE, San Sebastian, Spain; 3 CIBER-BBN, San Sebastian, Spain; 4 Medicina y Cirugía Experimental, Hospital General Universitario Gregorio Marañón, CIBERSAM, Madrid, Spain; 5 Department of Bioengineering and Aerospatial Engineering, Universidad Carlos III, Madrid, Spain; City of Hope National Medical Center and Beckman Research Institute, United States of America

## Abstract

Adult neurogenesis is restricted to specific brain regions. Although involved in the continuous supply of interneurons for the olfactory function, the role of neural precursors in brain damage-repair remains an open question. Aiming to *in vivo* identify endogenous neural precursor cells migrating towards a brain damage site, the monoclonal antibody Nilo2 recognizing cell surface antigens on neuroblasts, was coupled to magnetic glyconanoparticles (mGNPs). The Nilo2-mGNP complexes allowed, by magnetic resonance imaging in living animals, the *in vivo* identification of endogenous neural precursors at their niche, as well as their migration to a lesion site (induced brain tumor), which was fast (within hours) and orderly. Interestingly, the rapid migration of neuroblasts towards a damage site is a characteristic that might be exploited to precisely localize early damage events in neurodegenerative diseases. In addition, it might facilitate the study of regenerative mechanisms through the activation of endogenous neural cell precursors. A similar approach, combining magnetic glyconanoparticles linked to appropriate antibodies could be applied to flag other small cell subpopulations within the organism, track their migration, localize stem cell niches, cancer stem cells or even track metastatic cells.

## Introduction

In spite of new advances in understanding the biology of embryonic stem cells and induced pluripotent stem cells, tissue-specific stem cells remain the most promising cells for regenerative medicine, due to their ability to self-renew and differentiate into the distinct cell types that constitute a particular tissue. Neural precursors are mainly localized in the subventricular zone (SVZ) of the lateral ventricles and the subgranular zone (SGZ) of the hippocampus dentate gyrus [1]–[4]. In the adult SVZ, neural stem cells (B1 astrocytes) generate through different intermediates, neuroblasts and glial precursors, which differentiate into neurons and glia, respectively [1], [5]–[7]. It is known that neurogenesis in the adult brain plays an important role maintaining the homeostasis, such as in the olfactory bulb where a continuous supply of migrating neuroblasts is required for the generation of periglomerular interneurons. Indeed, neuroblasts migrate from the SVZ to the olfactory bulb through the rostral migratory stream (RMS) [8]–[12] as recently confirmed by magnetic resonance imaging (MRI) analyses of migrating endogenous neural cells with *in situ* endocytosed nanoparticles [13]–[17]. In addition, experiments using *in vivo* BrdU-labeled cells [18]–[21], or *in vitro* labeled cells subsequently grafted in a recipient brain [22]–[33] have shown that in response to brain insults, cells migrate towards the lesion site. MRI combined with contrast agents has been widely used as a noninvasive technique to study cell migration of grafted cells with an efficient labeling without impairment on cell survival, proliferation, self-renewal or multipotency [34]. Taken together, these data suggest migration of neural cells to damage sites, although without direct evidence for migration of any particular endogenous progenitor subpopulation, and allow envisaging the possibility that in response to brain damage there is neurogenesis in the adult brain.

To *in vivo* track an endogenous neural cell subpopulation migrating towards a brain damage site, we took advantage of the monoclonal antibody Nilo2, recognizing live neuroblast cells [35], which was coupled to recently developed magnetic glyconanoparticles (mGNPs) [36]. The Nilo2-mGNP conjugates were suitable for magnetic resonance imaging detection and were used to analyze *in vivo* neuroblast cell niches, as well as the migration of specifically labeled endogenous neuroblasts from their niche towards an astrocytoma lesion site.

## Materials and Methods

### Animals

Experiments were performed in compliance with the European Union and Spanish laws (Council Directive 86/609/EEC) and approved by the Committee of Animal Experimentation of the CSIC. For these experiments 6–8 week old FVB or C57Bl/6 animals, bred and housed in our animal facility under standard conditions were used. All surgery was performed under anesthesia, and efforts were made to minimize suffering.

### Antibodies

Nilo2 mAb was generated by the fusion of hamster B cells and the mouse myeloma X63Ag8, as described [35]. Purified Nilo2 was from Immunostep Inc. (Salamanca, Spain). Commercial antibodies and other reagents are described in Table 1.

**Table 1:** Commercial antibodies used in flow cytometry, immunocytochemistry, immunohistochemistry and immunoblotting.

PRIMARY ANTIBODIES								
Antibody	Host			Source	Clone or Cat. #		Ab Dilution	
SOX2	Rabbit, polyclonal			Chemicon	AB5603		1∶400	
DCX	Goat, polyclonal			Santa Cruz Biotech.	Sc-8066		1∶200	
PSA-NCAM	Mouse, monoclonal			Chemicon	MAB5324		1∶400	
PSA-NCAM	Mouse, monoclonal (IgM)			Abcys online	AbC0019		1∶1000	
CD4	Rat, monoclonal			BD Pharmingen	553043		1∶200	
CD4-FITC	Rat, monoclonal			BD Pharmingen	553055		1∶200	
CD8-FITC	Rat, monoclonal			BD Pharmingen	553031		1∶200	
CD11b	Rat, monoclonal			BD Pharmingen	553308		1∶100	
GFAP	Rabbit, polyclonal			Neomarkers	RB-087-A1		1∶200	
hup53	Rabbit, polyclonal			Cell Signalling Tech.	2521S		–	
CD3ε	Hamster, monoclonal			BD Pharmingen	553058		–	
**SECONDARY ANTIBODIES AND REAGENTS**								
**Antibody**		**Host**	**Source**			**Clone or Cat. #**		**Ab Dilution**
Anti-hamster IgG-HRP		Mouse	BD Pharmingen			554012		1∶5000
Anti-hamster-FITC		Mouse	BD Pharmingen			554011		1∶100
Anti-mouse IgG-Texas Red		Goat	Molecular Probes			T-862		1∶400
Anti-mouse IgM A546		Goat	Invitrogen			A-21045		1∶400
Anti-rat Alexa Fluor 647		Goat	Invitrogen			A-21247		1∶400
Anti-rabbit IgG-Cy3		Goat	Jackson ImmunoResearch			111-165-003		1∶400
Anti-goat 594		Chicken	Invitrogen			A21468		1∶400
Anti-hamster biotin		Mouse	BD Pharmingen			550335		1∶100
Streptavidin Alexa Fluor 647		–	Invitrogen			S32357		1∶400
Streptavidin Alexa Fluor 488		–	Invitrogen			S32354		1∶400
Streptavidin Texas Red		–	Invitrogen			S872		1∶400

### Synthesis and Characterization of the Protein G-magnetic Glyconanoparticles (mGNPs)

Water soluble magnetic glyconanoparticles consisting on a 4 nm magnetic core covered with a 1 nm gold shell coated with carbohydrates and an amphiphilic linker with an end-position carboxyl group were prepared and characterized as previously described [36]. Covalent immobilization through the carbonyl groups of a recombinant and commercially available protein G enables capture of IgG antibodies on the nanoparticles [37], [38].

Lyophilized nanoparticles (1.0 mg) were dissolved in 1 ml of PBS. The carboxyl groups were activated by adding a solution of N-ethyl-N′-(3-dimethylaminopropyl) carbodiimide hydrochloride (EDC) (40 µg, 0.21 mmol) and N-hydroxysuccinimide (NHS) (36 µg, 0.31 mmol). This mixture was shaken for 90 min, diluted to 3 ml of PBS and shaken for 3 hours at 10°C with protein G (161 µl, 1 mg/ml in PBS, Southern Biotech) on a 5∶1 (protein G: nanoparticles) ratio. The mixture was immediately centrifuged at 14000 × g for 90 min at 4°C. Uncoupled protein G was eliminated following washes in PBS. The resulting pellet was suspended in 25 mM Tris pH 9, 100 mM glycine. Protein G-glyconanoparticles (mGNPs) were characterized by MALDI-TOF spectrometry. The amount of coupled protein G was quantified by the Bradford method. Iron content was evaluated by induced coupled plasma optical atomic spectroscopy (ICP-OAS) estimating that 1.5 mg mGNPs contained 48 µg Fe. The relaxivity values *r_2_* of the mGNPs (137 mM^−1^s^1^ in PBS at 37°C, 1.4 Teslas) did not change in comparison to the precursor glyconanoparticles [36]. Core size of the protein G-glyconanoparticles, estimated from the TEM micrographs, was the same as those of the glyco-ferrites.

T2* estimation of mGNPs was calculated from phantom data. Phantoms were prepared on agar supplemented with copper sulfate with different concentrations of iron from dextran coated commercial nanoparticles Endorem™ (Guerbert Laboratories) or protein G functionalized nanoparticles (mGNPs). Measurements were carried out with a Bruker Biospec 70/20 scanner (7T) using a linear coil resonator, employing a multiple gradient echo sequence (parameters: TR 1500 ms, TE 4 to 53 ms, echo spacing 7 ms), from where the T2* map was calculated. Similar ROIS were traced on each image and their mean values were normalized to the background value. These values were represented versus iron concentration.

**Figure 1:**
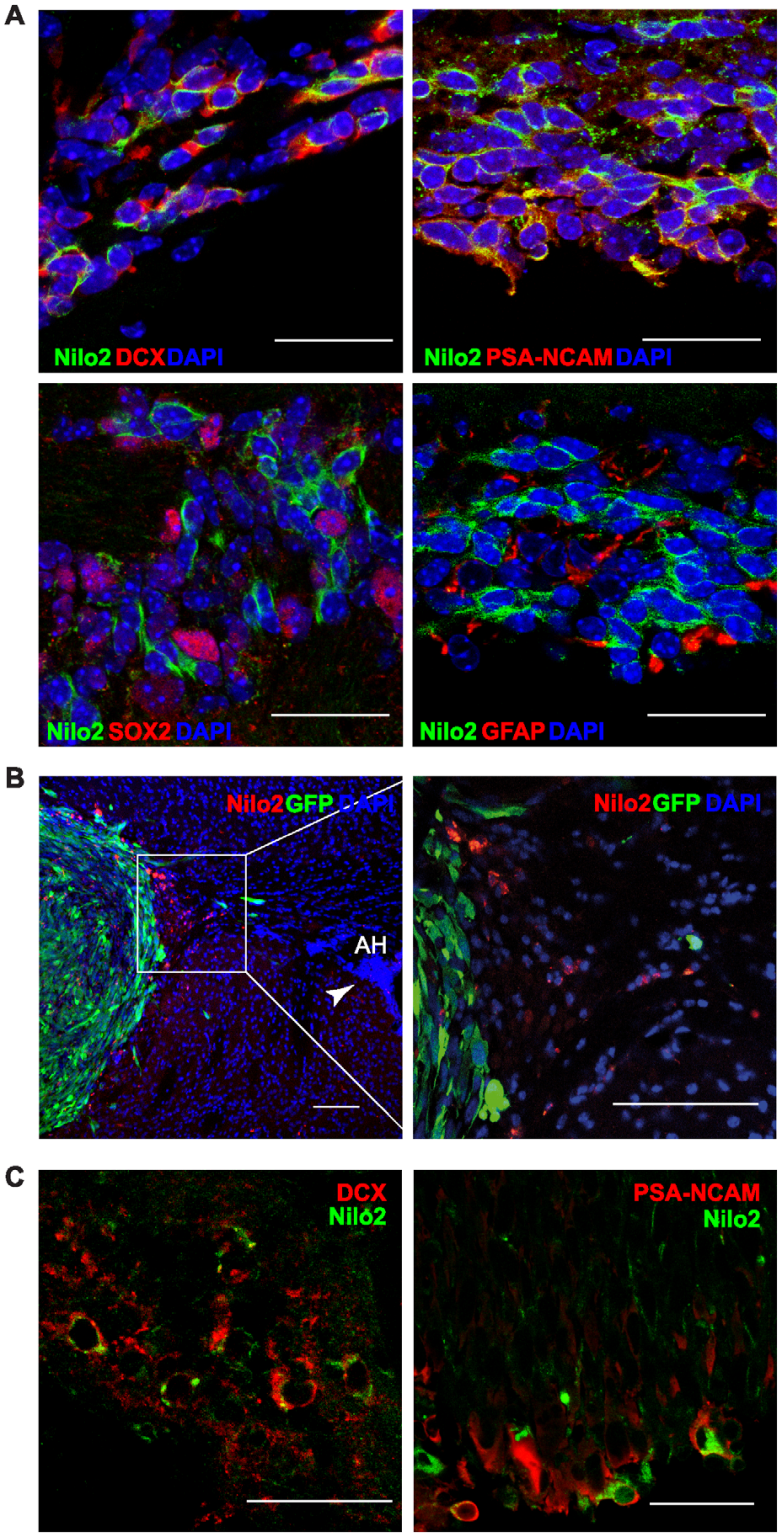
Identification of neuroblasts with Nilo2 monoclonal antibody. A , Immunohistochemistry of subventricular zone fixed brain sections double stained for Nilo2 (green) and DCX, PSA-NCAM, SOX2 or GFAP (red) detected by confocal microscopy. **B–C,** *In vivo* identification and characterization of Nilo2^+^ neural precursors surrounding a tumor induced after injection of CT-2A cells in the left *striatum*, following intraperitoneal injection one week latter of purified Nilo2 mAb, which was revealed in tissue sections with a secondary biotinylated anti-hamster Ig antibody and the appropriate fluorochrome. **B.** GFP- CT-2A cells were used (GFP^+^ in green), Nilo2^+^ cells were identified with streptavidin-Texas Red at low (left) and high (right) magnification. **C**, CT-2A cells were injected using the same protocol as in B. Nilo2^+^ cells in these tissue sections were identified with streptavidin-A488 (green) simultaneously with the neuroblast markers DCX or PSA-NCAM (red). Confocal microscopy analyses demonstrate that the Nilo2+ cells surrounding the tumor were DCX^+^ and PSA-NCAM^+^, indicating that these cells correspond to neuroblasts. DAPI was used to stain nuclei (blue). Scale bars: **A**, 25 µm; **B**, 100 µm; **C,** 45 µm; AH, anterior horn.

### Nilo2 Binding to Magnetic Nanoparticles

Protein G-magnetic glyconanoparticles (mGNPs) (100 µg) were incubated 5 h at 4°C with Nilo2 mAb (135 µg) in 0.1 M glycine buffer pH 9.0 on a final volume of 50 µl. After three PBS washes, nanoparticles were isolated by centrifugation and solubilized in 30 µl of PBS. The amount of Nilo2 mAb bound to the mGNPs was determined by western blot using SDS-PAGE and a secondary antibody antiHa-HRP (1∶5000). Purified Nilo2 mAb (0.75 µg) was used as standard and different volumes (1–3 µl) of the Nilo2-mGNPs suspension were loaded corresponding to 3.5–10.5% of the volume sample. Quantification by densitometry allowed to estimate that Nilo2-mGNPs contained 0.20 mg/ml Nilo2 (1.82 µg Nilo2/µg Fe).

Size and morphology of the Nilo2-mGNPs were estimated by transmission electron microscopy (TEM). Nilo2-mGNPs were dissolved in 20 mM Tris-HCl pH 7.4, 200 mM NaCl and applied during 1 min to a carbon-coated Cu-Pd grid with glow-discharge. After negative staining with 2% uranyl acetate for 40 s, samples were observed using a JEOL 1230 transmission electron microscope operated at 100 kV.

### Neurosphere Preparations and Cell Culture

Neurospheres for *in vitro* experiments were prepared from the SVZ of 6- to 8-week old FVB mice or from E13.5 C57Bl/6 olfactory bulbs as described [35]. Alternatively, for *in vivo* experiments with grafted cells, GFP^+^ neurospheres were obtained from β-actin EGFP transgenic mice (C57Bl/6 background) [39]. CT-2A mouse astrocytoma (gift from Prof. T.N. Seyfried, Boston, MA, USA), and GFP-CT-2A (gift from A. Martinez, I. Cajal, CSIC, Madrid, Spain) [40], were grown in RPMI medium, 10% heat-inactivated fetal bovine serum in 5% CO_2_ at 37°C and 95% humidity.

**Figure 2:**
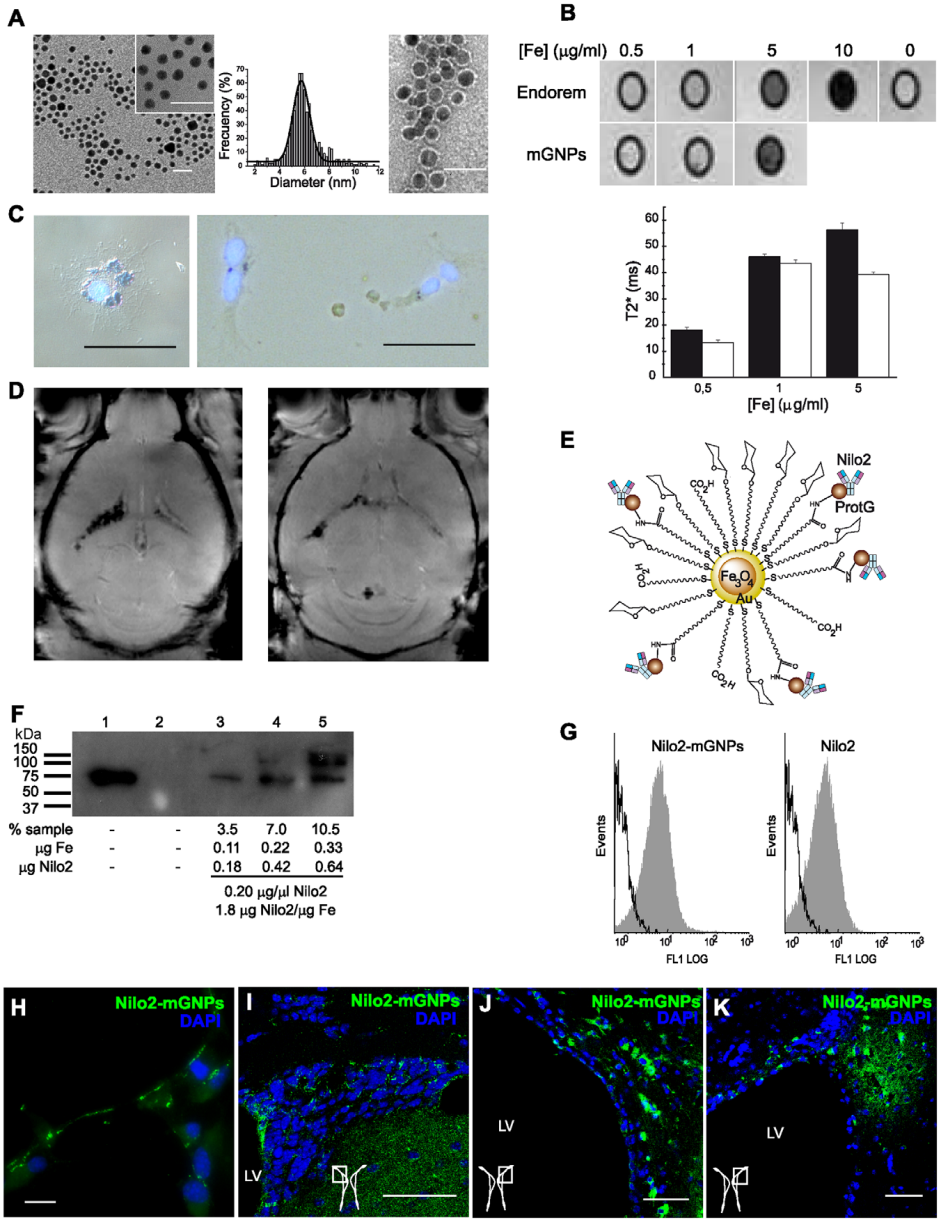
Physicochemical and functional characterization of Nilo2-mGNPs. A, Transmission electron microscopy (TEM) of mGNPs (left panel) or Nilo2-mGNPs (right panel) showing that both remained disperse in aqueous solution. Size distribution of glyconanoparticles before coupling to protein G was determined from TEM images. Data were fitted to a Gauss distribution (black line) (central panel). **B**, Phantoms from Endorem™ and mGNPs at different iron concentrations (top) allowed to estimate the transverse relaxation time constant T2* (bottom) represented as mean ± s.d. mGNPs (filled), Endorem™ (empty). **C**, Iron detection by Prussian blue staining on neurosphere cells preincubated with Endorem™ nanoparticles; 13×10^4^ cells with 50 µg/ml Fe, (left) or 3×10^4^ cells with 5 µg/ml Fe (right). **D**, Representative coronal MRI views from a mouse brain injected with iron-preloaded cells from (**C**) into the right lateral ventricle (low iron content) or into the left lateral ventricle (high iron content). The images correspond to two different slices from the same brain. **E**, Cartoon representing Nilo2-mGNPs. **F**, Western blot of Nilo2-mGNPs suspension used to estimate the amount of Nilo2 bound to mGNPs. Purified Nilo2 mAb (0.75 µg) was used as standard (lane 1). Different volumes (1–3 µl) of the suspension were loaded on lanes 3–5. Lane 2 was empty. The amount of Nilo2 mAb bound to the mGNPs was estimated by densitometry (1.82 µg Nilo2/µg Fe). **G**, Flow cytometry analyses of SVZ-derived neurosphere cells stained with Nilo2-mGNPs or Nilo2 alone, both revealed with a fluorescent secondary antibody. **H**, Fluorescence microscopy of Nilo2-mGNPs labeled neurosphere cells (green) grown in Matrigel. **I–K**, *In vivo* identification of neural precursors in mice intracranially injected with Nilo2-mGNPs (1 µl, 0.2 µg Nilo2 coupled to 0.11 µg Fe) either (**I**) contralaterally or (**J, K**) ipsilaterally to the injection site. Since Nilo2 antibody had already been injected *in vivo*, the brain sections were incubated with a secondary biotinylated anti-hamster Ig antibody and revealed with streptavidin-A488 (**H–K**) and analyzed by confocal microscopy. A scheme of the SVZ area analyzed is shown. DAPI was used to stain nuclei (blue). Scale bars: **A**, 25 nm; **C**, **H–K**, 50 µm. LV, lateral ventricle; AH, anterior horn.

### Intracranial Surgery

C57BL/6J mice were anaesthetized intraperitoneally with 100 mg/kg of ketamine and 10 mg/kg of xylacine. The mice heads were immobilized in a stereotaxic frame and intracranially injected with 1 µl of Nilo2-mGNPs in the *striatum* at coordinates +0.9 mm anterior, +0.75 mm lateral, −2.75 mm ventral from the bregma point. As control, PBS was used. Brain fixations were performed on anesthetized mice by transcardiac perfusion with 4% paraformaldehyde (PF) in 0.1 M phosphate buffer (fixation buffer). Brains were extracted and post-fixed overnight at 4°C in fixation buffer and cryoprotected in fixation buffer with 30% sucrose for two days at 4°C before freezing at −80°C. Fixed brains were cut in cryostat at 25 µm and slices maintained at −20°C in glycerol/etilenglycol buffer until analyzed.

*In vivo* identification of Nilo2^+^ cells in their niches was done injecting 1 µl of purified Nilo2 (0.25 µg) at stereotaxic coordinates +0.9 mm anterior, +0.75 mm lateral, −2.75 mm ventral refereed to bregma point. Mice (n = 4) were perfused 24 h later.

For neurosphere-Endorem™ graft experiments, neurospheres from E13.5 olfactory bulb embryos were disaggregated and incubated with Endorem™ nanoparticles (13×10^4^ cells with 50 µg/ml Fe or 3×10^4^ cells with 5 µg/ml Fe) for 48 h at 37°C. Collected cells were PBS washed and stereotaxically injected into the right (2 µl with 4×10^3^ cells from low iron content) or the left (2 µl with 3×10^4^ cells from high iron content) lateral ventricles at positions +0.14 anterior, +/−0.75 lateral, −2.25 ventral respect to bregma point. With these conditions the maximum iron injected was 0.22 µg Fe in the right or 17 µg Fe in the left lateral ventricles. Next day, MRI was performedon anesthetized mice (n = 2).

Tumor mice model was generated by intracranial graft of 10^2^−2×10^5^ CT-2A or GFP-CT-2A cells in 1 µl of PBS at stereotaxic coordinates +0.1 mm anterior, −2.25 mm lateral, −2.70 mm ventral into the right caudate putamen.

In GFP^+^-neurosphere graft experiments used to study migration kinetics of exogenous cells in response to an induced tumor, SVZ neurospheres (4×10^4^ cells) from adult EGFP-transgenic mice (n = 6) were injected at stereotaxic coordinates +0.14 anterior, +0.6 lateral, −2.25 ventral respect to bregma point (into the right lateral ventricle). CT-2A cells (1×10^4^ cells) were injected into the left striatum of mice (n = 3) three days later at position +0.1 mm anterior, −2.25 mm lateral, −2.70 mm to induce a tumor. Mice were sacrificed next day to analyze the location of GFP^+^ cells.

**Figure 3:**
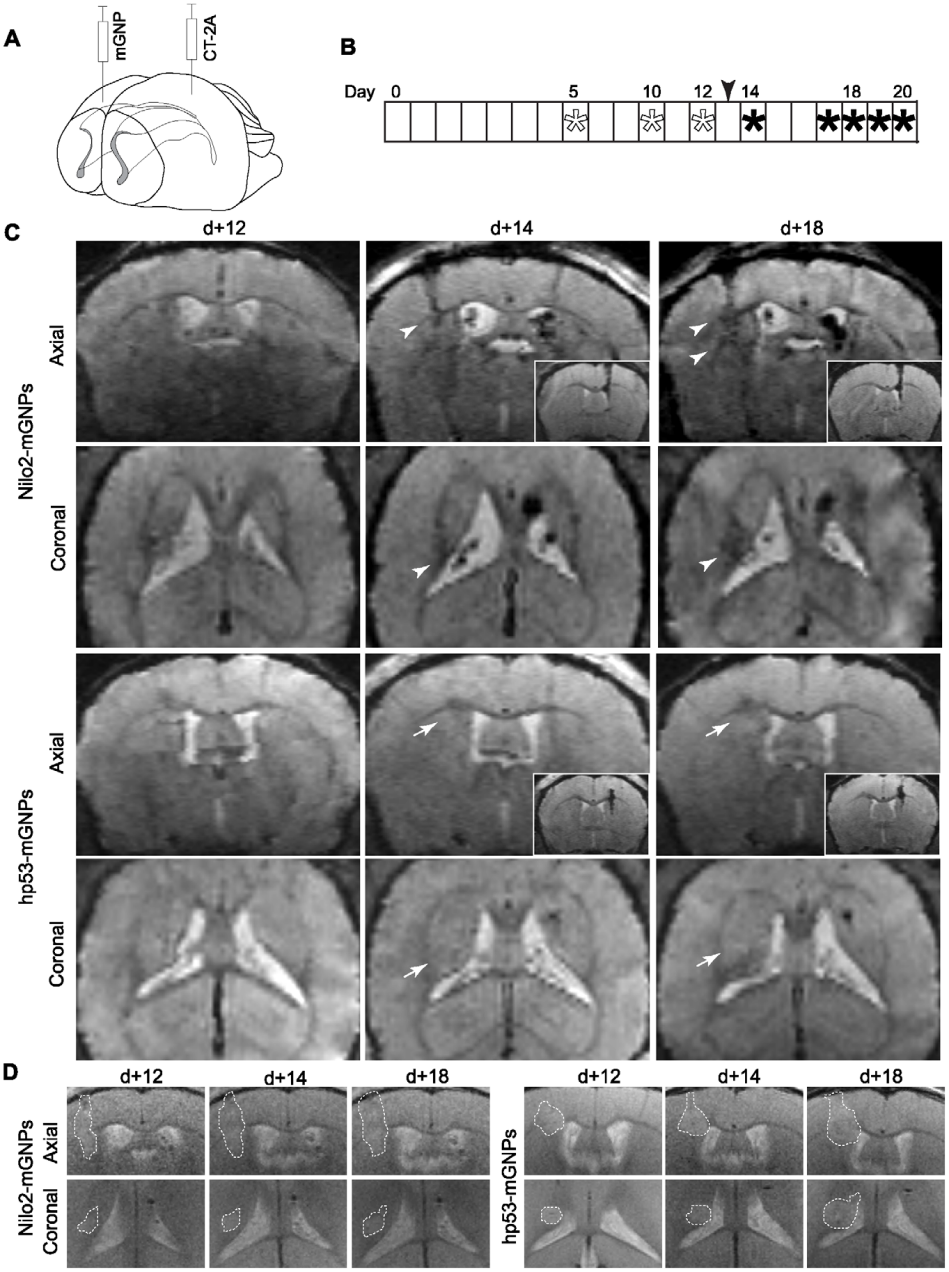
Nilo2-mGNPs allowed to reveal MRI hypointense signals in brain damage sites. **A**, Schematic representation of the injection sites in the brain for the CT-2A astrocytoma cells (left hemisphere, d 0) and Nilo2-mGNPs (contralaterally in a more rostral position). **B**, Experiment schedule, Nilo2-mGNPs injection day is indicated with an arrowhead, MRI acquisitions are shown with asterisks, before (empty) or after nanoparticle injection (filled). **C**, Representative MRI analyses of mice injected with GFP-CT-2A cells (d 0) and either Nilo2-mGNPs (n = 4), or anti-human p53-mGNPs (n = 4). Axial and coronal views on the day before (d+12), or either one (d+14) or five (d+18) days after nanoparticle injection. Arrowheads indicate the hypointense signals at the tumor site. Arrows indicate the absence of hypointense signals in hp53-mGNPs injected mice at the tumor location. Mice were sacrificed on day d+20. Insets in axial views from hp53-mGNPs and Nilo2-mGNPs injected mice show the signals due to nanoparticles at the injection site. **D**, MRI tumor localization. T2 acquisitions showing the tumor localization on sections corresponding to the T2* MRI images shown in (**C**). Tumor sites, defined by hyperintense signals are surrounded by a dotted line (white).

**Figure 4:**
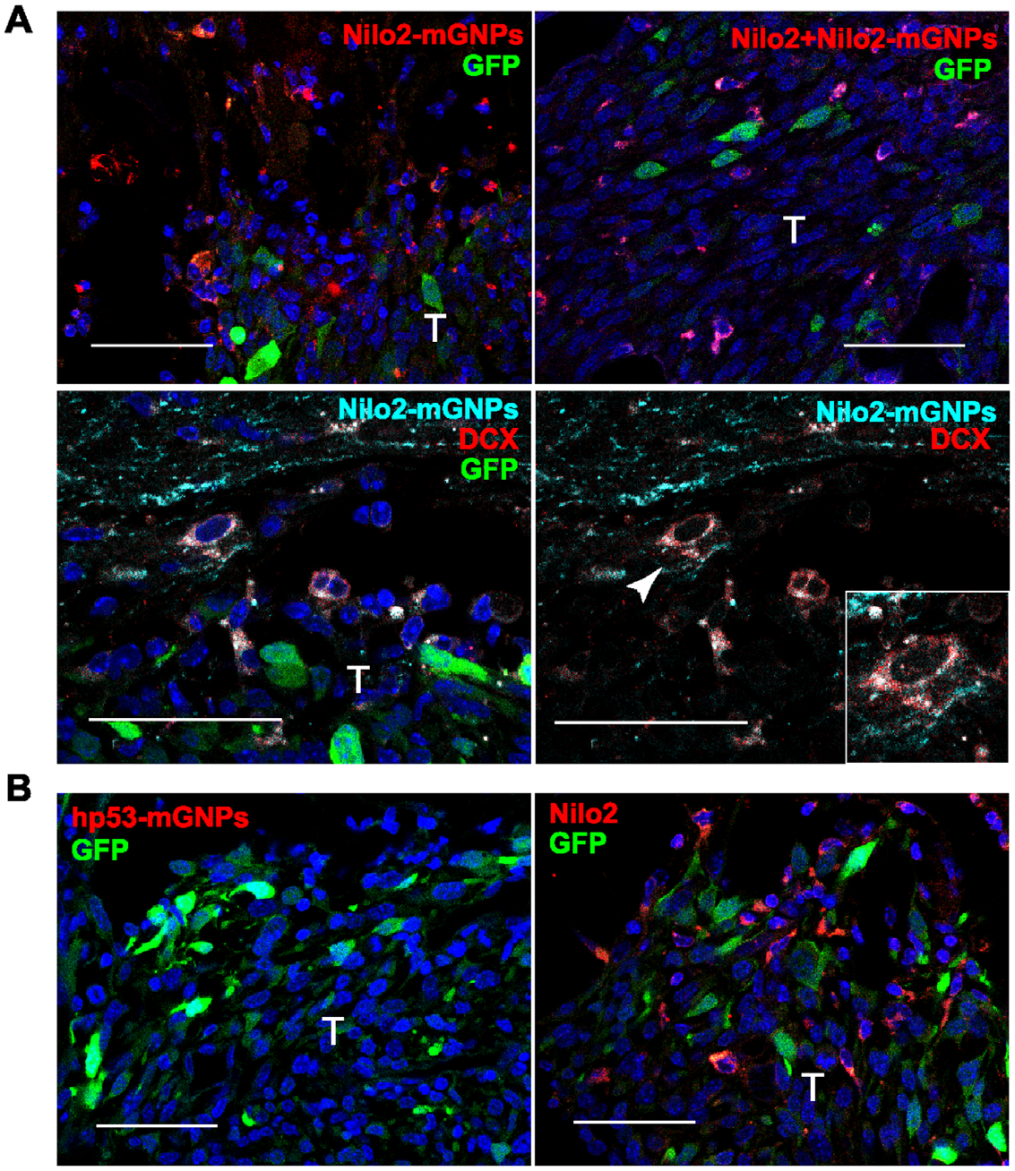
Hypointense MRI signals at the tumor site correspond to neuroblasts. Immunohistochemical analyses of fixed brains from the mice analyzed in Figure 3. **A**, Tissue sections from animals injected with Nilo2-mGNPs incubated with a fluorescent secondary antibody (left) or with additional Nilo2 mAb (right) did not show differences neither in the number nor in the intensity of labeled cells (top). Confocal analyses confirmed that the hypointense signals in the tumor site (T, GFP^+^ cells) correspond to neuroblasts (Nilo2^+^DCX^+^) (bottom). Inset, detail of the cell labeled by the arrowhead. **B**, Specificity was shown on hp53-mGNPs injected mice using a fluorescent secondary anti-human p53 mAb, which did not reveal migration of hp53-mGNPs-labeled cells (left), although Nilo2^+^ cells migrated towards the tumor (GFP^+^ cells) (right). Scale bars: 50 µm.

### Magnetic Resonance Imaging

MRI studies were performed in a Bruker Biospec 70/20 scanner using a combination of a linear coil (for transmission) with a mouse head phase array coil (for reception). Animals were anesthetized with sevofluorane (5% for induction and 2% for maintenance) and placed in an MRI-adapted stereotaxic holder. Respiration and body temperature were continuously monitored. MRI acquisition protocol included an initial flash sequence (repetition time: 100 ms, echo time: 6 ms, field of view: 4 cm, matrix: 128×128) to center the Field of View (FOV), followed by a shimming procedure applied to a region of interest covering the head (FOV = 3×2×2 cm, matrix = 64×64×64) and based on a Field Map sequence (TR = 20 ms, TE = 1.43 and 5.42 ms).

As an anatomical reference we used a T2-weighted coronal image (TR = 2500 ms; TE, 33 ms; α = 180°; FOV = 2×2 cm; matrix = 256×256; slice thickness = 0.5 mm) and nanoparticles were detected and tracked with a 3D multi gradient echo (MGE) sequence (TR = 200 ms; 8 echoes, TE = 10 to 45 ms; echo spacing = 5 ms; α = 15°; FOV = 1.6×1.6×1.5 cm; matrix = 192×96×96).

To increase the signal-to-noise ratio (SNR) and improve image contrast, the different echo images were added (in magnitude). To display the results, the tumor area was manually segmented on the T2 scans and spatially aligned with the MGE image.

**Figure 5:**
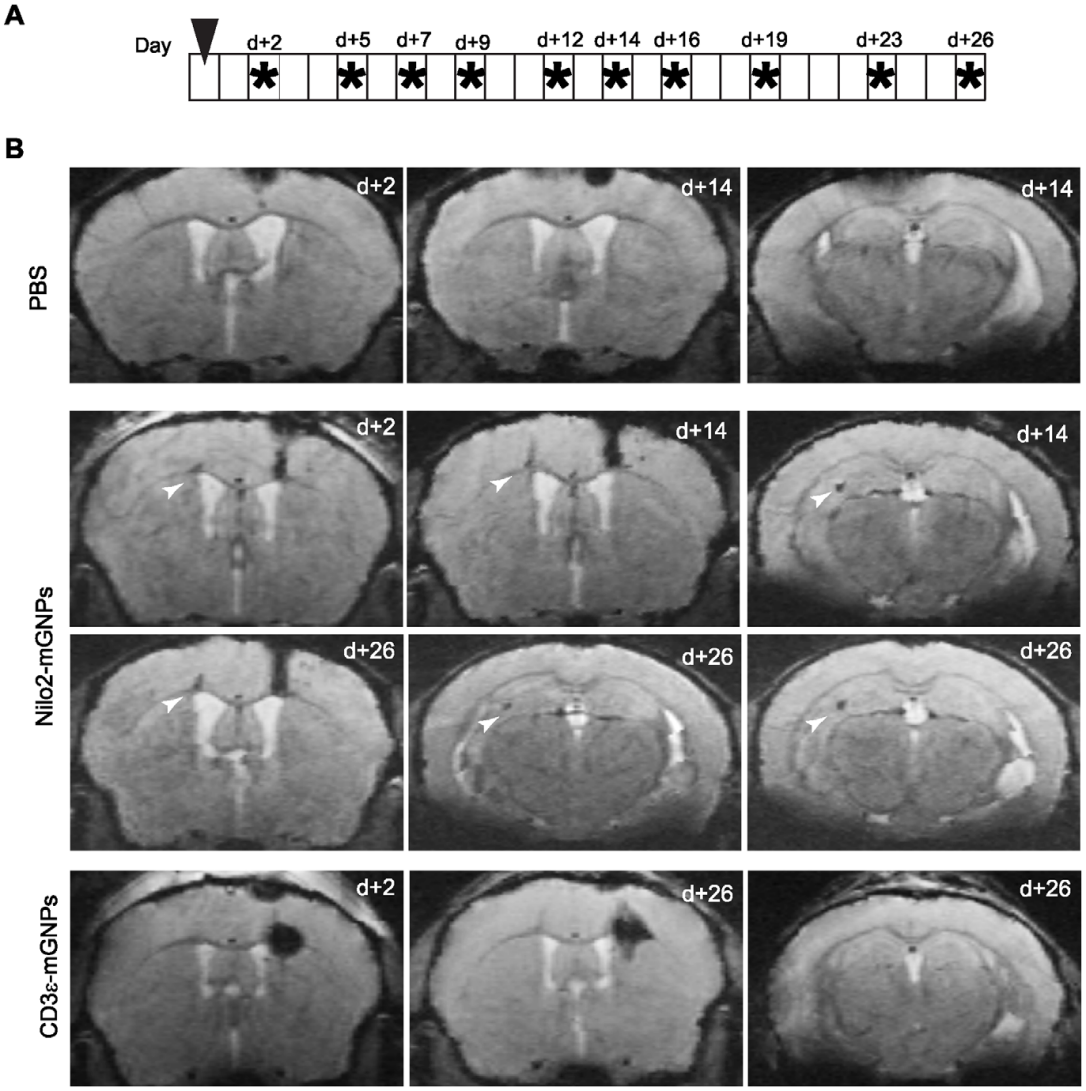
Nilo2-mGNPs identify neuroblasts in the main neurogenic niches. A , Experimental schedule, Nilo2-mGNPs injection day is indicated with an arrowhead, MRI acquisition days are shown with asterisks. **B**, Axial views at different rostrocaudal positions of a representative MRI at different time points after the injection of PBS (1 µl, n = 2), Nilo2-mGNPs (n = 2) or CD3ε-mGNPs (n = 2) into the right hemisphere in the absence of tumor induction. Arrowheads indicate the hypointense signals in neuroblasts niches.

**Figure 6:**
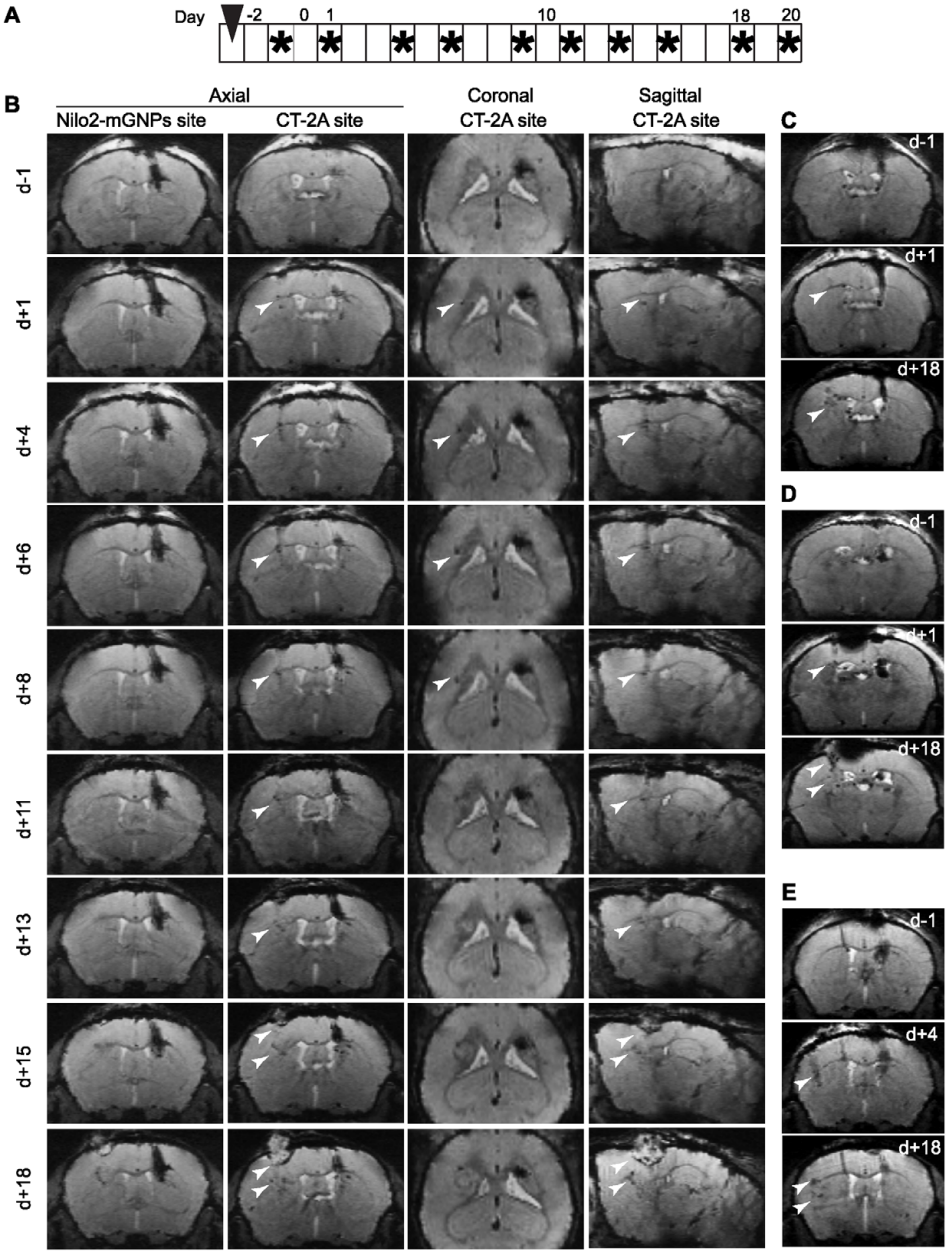
Migration kinetics of Nilo2-mGNPs labeled cells in response to damage. A , Experimental schedule, Nilo2-mGNPs injection day is indicated with an arrowhead, MRI acquisition days are shown with asterisks, day 0 represents the day in which the CT-2A injury was generated. Asterisk on day 20 indicates an *ex-vivo* MRI of fixed brain before immunohistochemical analysis. **B**, Axial, coronal and sagittal views of a representative MRI one day before (d−1), or one (d+1) to18 (d+18) days after the injection of tumor cells in the left hemisphere (n = 6) showing the Nilo2-mGNPs injection site (Nilo2-mGNPs site) and CT-2A injection site (CT-2A site). Arrowheads indicate the hypointense signals detected at the tumor vicinity. **C–E**, Axial MRI acquisitions from three additional mice, the day before (d−1), one (d+1), four (d+4) or 18 (d+18) days after tumor cell injection.

### Immunological Analyses and Staining Procedures

Neurosphere adherent cultures were performed on Matrigel Basement Membrane Matrix Growth Factor Reduced (BD Pharmingen) pre-coated coverslips with 1∶20 dilution in culture media. Cells were fixed with 4% PF in PBS buffer for 15 min at RT. Quenching was performed by adding 0.1 M glycine pH 7.4 for 15 min at RT. After three PBS washes, blocking was performed by incubating the coverslips with 10% mouse serum in PBS during 1 h at RT. Fixed cells were incubated overnight with Nilo2-nanoparticles (5 µl/well) mAb at 4°C. After three PBS washes, cells were incubated with the secondary antibody (anti-Ha-FITC 1∶100, or anti-Ha-Cy5.5 1:500) for 1 h.

To assess whether MRI signals corresponded to labeled neuroblasts, mouse brains from animals used in MRI analyses were fixed and cryopreserved for cryostat sectioning in a parallel plane to that of axial MR imaging. Serial 20–25 µm thick frozen sections were collected through the entire mouse brain. Anatomical landmarks such as corpus callosum, anterior commisure of the brain and lateral ventricles opening and shape, were used for the spatial alignment of MRI and immunohistochemical sections.

Brain sections of mice intracranially injected with Nilo2 or with Nilo2-mGNPs were blocked with 10% mouse serum in PBS during 1 h at RT and stained with anti-Ha-FITC (1∶100). Alternatively, anti-Ha biotin (1∶100) and streptavidin-A488 (1∶400) or streptavidin-Texas Red (1∶400) were used to amplify the signal. Coverslips were mounted in DAPI/Mowioll and analyzed in a Nikon Eclipse 80 i fluorescence microscope or a LEICA TCS-SP2-AOBS confocal microscope.

For *in vivo* identification of Nilo2^+^ cells surrounding the brain tumor, mice were intraperitoneally injected with Nilo2 ascites at 10 µg/g of body weight one week after the stereotaxic injection of 100 CT-2A cells. Next day, mice were euthanized and 25 µm sections of fixed brains were analyzed using Cy5.5-labeled anti-hamster antibody.

In neurosphere cultures incubated with Endorem™, iron from nanoparticles was reduced with acid solution of potassium ferrocyanide and the blue pigment formed (Prussian Blue) was detected with bright field microscopy.

### Flow Cytometry

Single cell suspensions from neurospheres were obtained by mechanical disaggregation. Unspecific antibody binding was blocked with PBS, 10% mouse serum, 3% BSA, 0.0025% NaN_3_ for 30 min at 4°C. An excess of Nilo2-mGNPs coupled to nanoparticles was added to the cell suspension and incubated for 1 h at 4°C. Cells with Nilo2 undiluted hybridoma were incubated as a control. After PBS washes, cell suspensions were stained with anti-Ha-FITC (1∶100 diluted in PBS, 5% BSA, 0.025% NaN_3_). Following additional PBS washes, cells were resuspended in 300 µl cold PBS until FACS measurements (Epics XL, Coulter). Propidium iodine was added (25 µg/ml) to each sample to gate on living cells.

**Figure 7:**
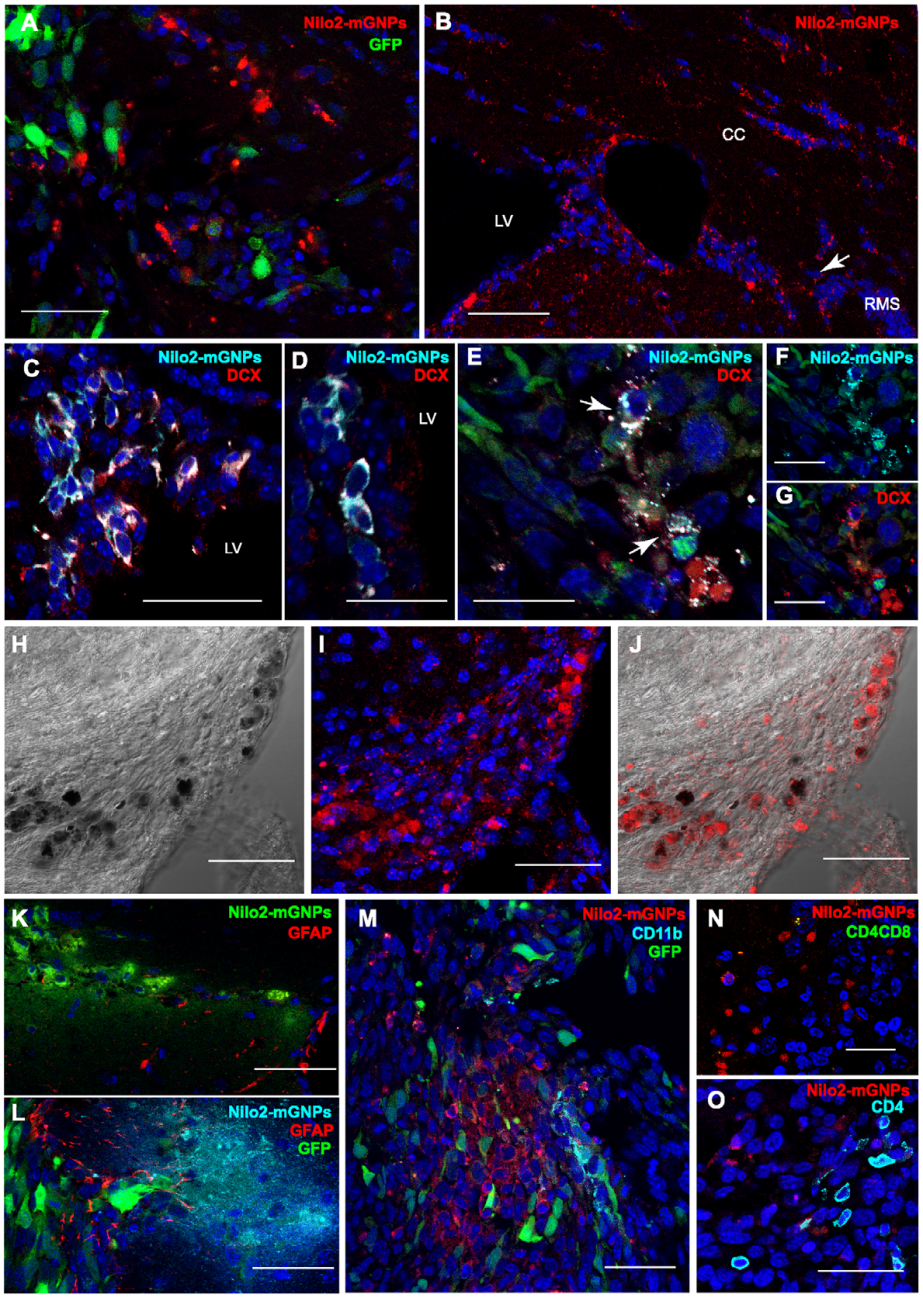
Nilo2-mGNPs specifically label Nilo2^+^ neuroblasts migrating towards the tumor site. **A, B**, Immunohistochemical analyses of the brain from Figure 6B with a fluorescent secondary antibody demonstrating the migration of Nilo2^+^ cells (red) towards the tumor area (GFP^+^) (**A**), or migrating from the contralateral ventricle through the RMS and the CC (**B**). **C–G**, The complexes Nilo2-mGNPs targeted Nilo2^+^ DCX^+^ cells in the contralateral ventricle to the tumor site (**C**), in subependymal positions of the lateral ventricles (**D**) or surrounding the GFP-CT-2A tumor (green) (**E–G).** **H**, Bright field microscopy showing nanoparticles in black. **I**, Nilo2 mAb coupled to mGNPs was revealed with a fluorescent secondary antibody (red). **J**, Merge from (**H**) and (**I**) indicating that Nilo2 mAb was still coupled to the mGNPs 23 days after intracranial injection. **K–O**, Nilo2-mGNPs were not engulfed by astrocytes (GFAP^+^), microglia (CD11b^+^) nor T lymphocytes (CD4^+^, CD8^+^). Mice were injected with Nilo2-mGNPs (**K**) in the absence or (**L–O**) in the presence of GFP-CT-2A tumor cells (green). DAPI was used to stain nuclei. Scale bars: **A, B**, 50 µm; **H–M, O, C**, 45 µm; **N, D–G**, 20 µm. LV, lateral ventricle; CC, *corpus callosum*; RMS, rostral migratory stream.

## Results

### Nilo2 Coupled to Magnetic Nanoparticles Identified *in vivo* Neuroblasts in Neurogenic Niches

The monoclonal antibody Nilo2, recently developed and characterized in our laboratory, was chosen for this study since it identifies, in living cells surface, antigens on subventricular zone (SVZ) neural precursors [35]. In fixed brain sections, double stainings revealed that Nilo2^+^ cells were positive for neuroblast markers and negative for neural stem cell markers (DCX^+^, PSA-NCAM^+^, GFAP^−^ or SOX2^−^) (Figure 1A). To ascertain whether Nilo2 could be used for the *in vivo* identification of neuroblasts at their niches, Nilo2 (0.25 µg) was intracranially injected into the right striatum, mice were sacrificed 24 h after and the fixed tissue was incubated with a secondary antibody recognizing hamster IgG, to reveal binding of Nilo2 mAb. These experiments allowed the identification of Nilo2^+^ cells either in the ipsi- or the contralateral SVZ, with respect to the injection site (data not shown), demonstrating that Nilo2 can identify its specific antigens *in vivo*. In addition, stereotaxic transplantation of mouse GFP^+^CT-2A cells generated an astrocytoma at the graft site in few days [40], where the tumor cells could be identified as GFP^+^. Taking advantage of the blood-brain barrier breakdown induced by the brain tumor [41], Nilo2 mAb was intraperitoneally injected one week after grafting the GFP^+^CT-2A cells and the animals were sacrificed by transcardiac perfusion 24 hours later. Staining of fixed brain tissue sections with an anti-Nilo2 fluorescent-labeled secondary antibody revealed that the growing tumor mass was surrounded by Nilo2 positive cells (Figure 1B). These cells were further characterized as *bona fide* type I neuroblasts (neural progenitors) since they were also positive for DCX and PSA-NCAM (Figure 1C). Appearance of Nilo2^+^ cells surrounding the tumor was concomitant with a strong reduction in Nilo2 staining of the contiguous anterior horn of the lateral ventricle (white arrowhead, Figure 1B). The presence of Nilo2^+^ cells at the damage site, away from their niche, opened up the possibility to use this mAb as a marker for *in vivo* analyses of endogenous neuroblasts in pathophysiological conditions.

To explore the *in vivo* behavior of endogenous neuroblasts in response to local brain damage, Nilo2 monoclonal antibody was coupled to magnetic glyconanoparticles (Nilo2-mGNPs). This approach allowed the *in vivo* identification of neuroblasts, following their migration by magnetic resonance imaging (MRI). The magnetic glyconanoparticles were composed of a magnetite core covered by a gold shell bearing sugars and carboxyl-ending linkers [36], to which protein G was coupled, allowing them to bind antibodies (Figure 2). Magnetic glyconanoparticles were characterized by transmission electron microscopy before and after protein G coupling, measuring the size distribution (∼6 nm diameter) (Figure 2A). The Nilo2-mGNP complexes were highly stable, monodisperse nanostructures with magnetic properties comparable to commercial nanoparticles as revealed by phantom analyses (Figure 2B). The minimal iron concentration that could be detected with our MRI equipment was estimated from phantom data as 0.5 µg/ml. Neurospheres pre-loaded with 50 or 5 µg/ml iron using Endorem™ nanoparticles (Figure 2C) were grafted intra ventricles and subsequently detected by MRI, with different hypointensities, depending on the iron concentration (Figure 2D). From these data, a detection limit of ≤0.22 µg of iron (right hemisphere) was estimated. Complexes Nilo2-mGNPs contained 0.11 mg/ml of iron and 0.20 mg/ml of Nilo2 and the mAb retained its specificity, since they identified *in vitro* cells from neurosphere cultures (Figure 2E–H). In addition, Nilo2-mGNPs identified *in vivo* Nilo2^+^ neural precursors in their neurogenic niches after intracranial injection into the right hemisphere (1 µl of Nilo2-mGNPs containing 0.20 µg Nilo2 and 0.11 µg iron) (Figure 2 I-K) as uncoupled Nilo2 (0.25 µg, 1 µl) did. This was shown by staining fixed brain sections from these mice sacrificed one day after surgery, with an appropriate secondary antibody.

**Figure 8:**
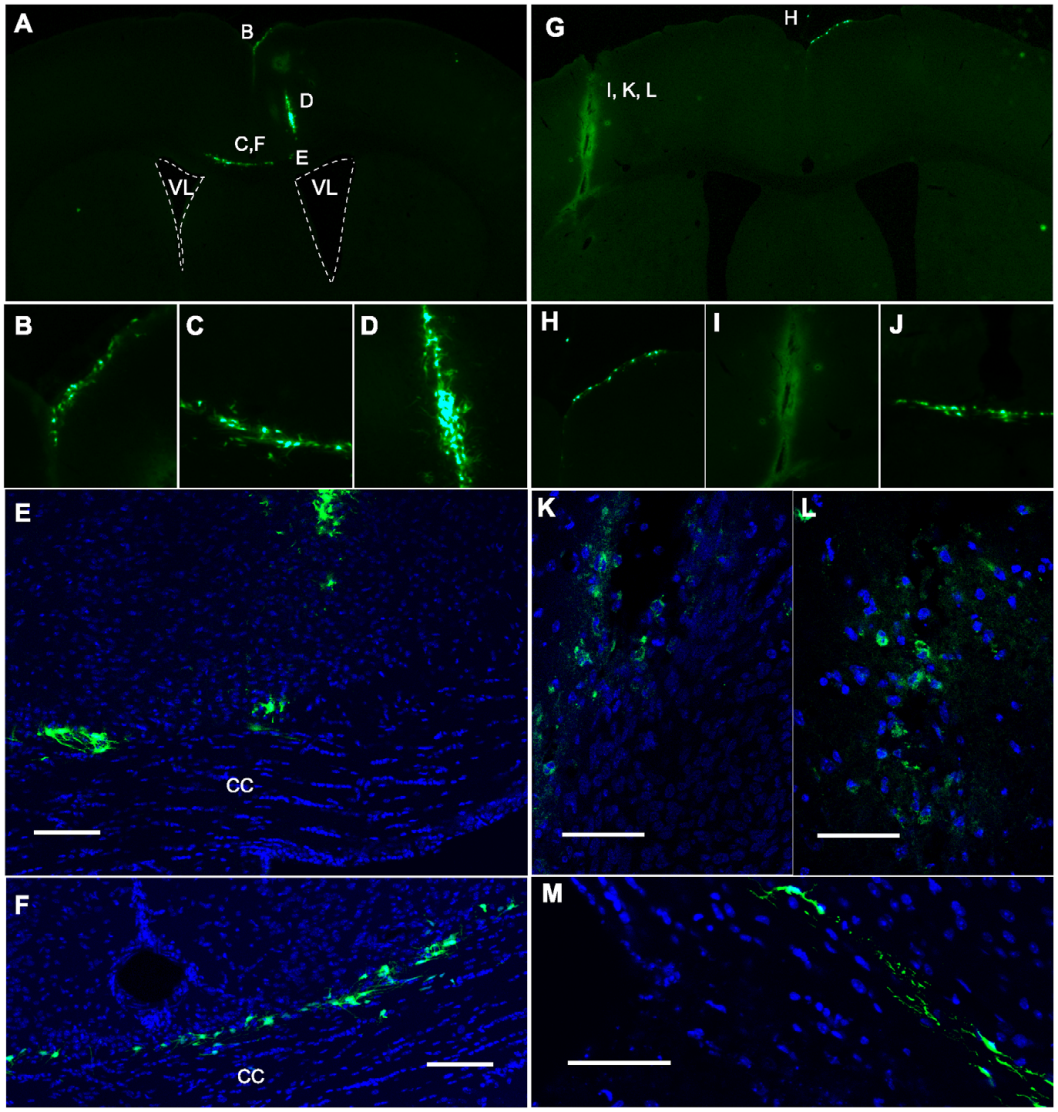
Exogenous GFP^+^ neural precursors migrated to the damage site in less than 24 hours. A–F , control or **G–M**, CT-2A injected animals were subsequently injected by stereotaxic surgery with SVZ-derived GFP^+^-neurosphere cells in the contralateral *striatum*. Mice were sacrificed 24 hours after injection of the CT-2A cells and brain tissue sections were analyzed by either (**A–D**, **G–J**) stereo microscopy or (**E, F, K–M**) confocal microscopy. Migrating GFP^+^ cells in (**B, H**) cortex; (**D, E**) injection site of neurosphere cells; (**C, E, F**) in CC; (**I, K, L**) injection site of tumor CT-2A cells; (**J, M**) CC in a caudal position respect to both injection sites. Scale bars: **E, F**, 150 µm; **K, L**, 50 µm; **M**, 75 µm.

**Figure 9:**
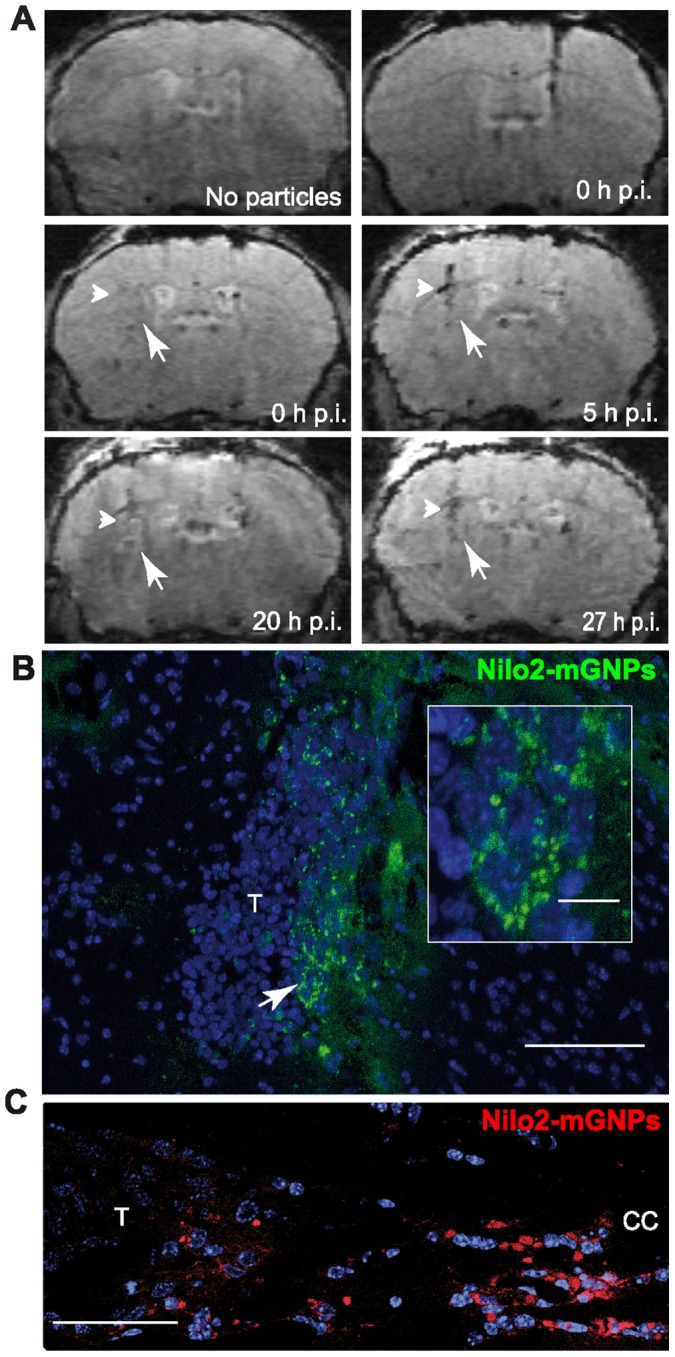
Endogenous Nilo2^+^ cells migrate fast and orderly in response to a tumor. Nilo2-mGNPs were injected into the right hemisphere and CT-2A cells into the left *striatum* four days later (n = 8). **A**, Nilo2^+^ cell migration was analyzed using MRI before (0 h), 5 h, 20 h and 27 h after CT-2A injection. Arrowhead and arrow on each panel show the positions of the hypointense signals after injection of the tumor cells (5 h, 20 h and 27 h), and the lack of hypointense signals before injection of the tumor cells (0 h). Control MRI analyses before the injection of the Nilo2-mGNPs (no particles) and injection site of these complexes at 0 h were included. **B, C**, Immunohistochemical analyses were performed after the last MRI at 27 h. Some of the migrating cells had already reached the tumor injection site (T), whereas others were still migrating through the CC. Nuclei were stained with DAPI (blue). White arrow in (**B**) indicates the magnified region in the inset. Scale bars: **B**, 75 µm; inset in **B**, 10 µm; **C**, 50 µm.

### Brain Tumors Induce Endogenous Neuroblast Migration

The CT-2A astrocytoma was chosen as a model due its high reproducibility on tumor development. GFP^+^CT-2A cells were grafted into the mice brain left hemisphere by stereotaxic surgery. These cells generated a tumor over the following 10–12 days (Figure 1B, and Figure 3). At this time point, Nilo2-mGNPs were stereotaxically injected in a more rostral position of the right hemisphere and contralaterally to the tumor site, without any detectable ventricle damage. Comparison of the T2* MRI measurements the day before (d+12) and the day after Nilo2-mGNPs injection (d+14) showed the appearance of hypointense signals (black dots) at the tumor graft site (arrowheads in Figure 3C), which were undetectable before nanoparticles injection (d+12), although at this time-point the T2 images indicated that the tumor was already formed (Figure 3D). In addition, hypointense signals also appeared in both the cerebrospinal fluid and the lateral ventricle walls (Figure 3C, d+14). These hypointense signals increased in the region surrounding the damage site with time. Black spots in the left lateral ventricle (d+14 in Figure 3C) reached the tumor site (arrowhead, coronal view on Figure 3C) four days later (d+18 in Figure 3C), indicating an accumulation of Nilo2-mGNPs at the tumor site concomitant with tumor growth (hyperintense signals in Figure 3D). The hypointense signals detected by MRI at the nanoparticles injection site show that both Nilo2-mGNPs and hp53-mGNPs were injected at the striatum (insets Figure 3C). The nanoparticles were not in direct contact with the cerebrospinal fluid, thus minimizing the passive diffusion effects. A control mAb (anti-human p53) coupled to the same mGNPs failed to generate a signal at the tumor vicinity (arrow) or within the ventricles, although the T2 MRI signals demonstrated that the tumor was formed (Figure 3C, D). Unlike Nilo2-mGNPs, hp53-mGNPs were exclusively detected at the injection site (Figure 3C), indicating that the migration specificity of the Nilo2-mGNPs was given by the Nilo2 mAb. To confirm that the MRI signals corresponded to Nilo2-mGNPs, mice were then sacrificed (d+20 in Figure 3B) and the fixed brain sections directly labeled with an anti-Nilo2 secondary fluorescently labeled antibody. These analyses allowed to verify that the MRI hypointense signals surrounding the tumor site corresponded to Nilo2^+^ cells (Figure 4A). The Nilo2^+^ cells surrounding the GFP-tumor were also DCX^+^ cells (Figure 4A), indicating that they correspond to *bona fide* neuroblasts. These results have been confirmed by additional stainings of Nilo2-mGNPs *in vivo* together with DCX (see below Figure 7C–G). When these tissue sections were incubated with additional Nilo2 mAb, we failed to detect an increase of the number of Nilo2^+^ cells, suggesting that most of the neuroblasts were already labeled with Nilo2-mGNPs. Immunohistochemical analyses corroborated that hp53-mGNPs remained undetectable either at the vicinity of the tumor or scattered through the parenchyma (Figure 4B), although it was still detected by MRI at its injection site (Figure 3C). It should be noted that in the hp53-mGNPs injected animals, neuroblasts surrounded the tumor as shown by the staining with Nilo2 mAb (Figure 4B).

Aiming to favor recognition of the neuroblast niches by the Nilo2-mGNPs, the immunonanoparticles were injected into right SVZ before tumor induction, without any detectable ventricle damage. Indeed, in these mice hypointense signals in the principal neurogenic niches were detected, which were stable during 26 days in the absence of tumor (Figure 5). Conversely, mice injected with the same volume of PBS failed to produce any hypointense signal due to hemorrhage (Figure 5B, d+2 to d+14). Specificity was corroborated using mGNPs coupled to an isotypic control anti-mouse CD3ε hamster mAb, where the detectable MRI signals were restricted to the SVZ injection site, even 26 days after injection (Figure 5B, d+2 to d+26). These data allowed to discard that appearance of hypointense signals surrounding the tumor in Nilo2-mGNP injected animals were due to passive diffusion of the nanoparticles. In the presence of tumor induction due to the graft of CT-2A cells, 72 h after the injection of Nilo2-mGNPs, results comparable to the ones shown in Figure 3 were obtained (Figure 6A). Indeed, in these experiments, Nilo2-mGNPs labeled the neuroblast niches, as demonstrated by MRI analyses preceding the graft of the tumor cells. Furthermore, hypointense signals near the damage site were detected by MRI as soon as 24 h after tumor injection (arrowheads, Figure 6, d+1). These hypointense signals increased and accumulated surrounding the tumor (Figure 6B, C) and corresponded to endogenous Nilo2^+^ DCX^+^ cells (Figure 7). These cells migrated from the lateral ventricles through different structures including the highly dense *corpus callosum* (CC) (Figure 7A, B). In addition, Nilo2-mGNPs identified bona-fide neuroblasts in subependymal patches on the lateral ventricle and the anterior horn of the contralateral ventricle with respect to tumor localization, since all the Nilo2^+^ cells were also DCX^+^ (Figure 7C–G). Immunohistochemical analyses on fixed brain sections from MRI-analyzed animals corroborated that Nilo2 mAb remained coupled to the mGNPs *in vivo* for at least 23 days, as shown by the co-localization of the nanoparticles with Nilo2 (Figure 7H–J). In tissue sections from these animals, Nilo2-mGNP^+^ cells were co-stained with specific antibodies recognizing glial cells (GFAP), activated microglia cells (CD11b) or T lymphocytes (CD4, CD8). The absence of double stained cells allowed to formally exclude that the hypointense signals surrounding the tumor were due to cells that, after unspecifically engulfing the Nilo2-mGNPs, migrated to the damage site (Figure 7K–O), in full agreement with the experiments using hp53 or CD3ε control antibodies coupled to the mGNPs (see above).

### Migration of Neural Precursors Starts in a Few Hours, is Fast and Orderly

From these MRI experiments, migration rates were estimated in 150 µm/h, and were equivalent to the migration rates (less than 24 h) detected for exogenous GFP^+^-neurosphere-derived cells following brain injury (Figure 8). The position of the tumor graft determined the time for Nilo2-mGNP-labeled cells to reach the damage site, increasing in grafts localized in more lateral positions (Figure 6E, d+4). Further MRI analyses on animals where Nilo2-mGNPs were injected before contralaterally grafting CT-2A tumor cells revealed the detection of the first hypointense signals in the tumor region as early as five hours after graft injection, resulting on an estimated migration rate of 700 µm/h. These signals were maintained for at least 27 hours, where the presence of Nilo2^+^ cells around the CT-2A tumor was confirmed by immunohistochemistry (Figure 9). We also detected additional Nilo2^+^ cells in the *corpus callosum* and the area between this structure and the tumor (Figure 9C). Since Nilo2^+^ cells in *wild-type* animals are mostly restricted to their niche in the lateral ventricle [35], their presence in the highly dense *corpus callosum*, together with their morphology with elongated nuclei and cytoplasmic projections [42], [43], reinforce the notion that these cells represent actively migrating neuroblasts from their niche towards the damage site induced by the tumor.

## Discussion

The monoclonal antibody Nilo2 was described as recognizing surface antigens from mouse type I neuroblasts and in immunohistochemistry identifies cells within the anterior horn of the lateral ventricle [35]. We have substantiated these data and in addition we show that Nilo2 mAb is able to identify these cells *in vivo*, following intracranial injection of the antibody. We confirmed the identity of the labeled cells *in vivo* by immunohistochemistry with other neuroblast type I markers such as DCX and PSA-NCAM. Our data also show that following the local injection of CT-2A cells, which generated an astrocytoma at the graft site, Nilo2^+^ cells could be detected surrounding the tumor. As the detection of Nilo2^+^ cells surrounding the tumor was concomitant with a strong reduction in their number in the contiguous lateral ventricle niche, suggested that Nilo2^+^ cells might migrate from their niche towards the tumor graft site. This allowed us to envisage the possibility of using this mAb as a marker for endogenous neuroblast migration analyses in living animals. The concept of endogenous neuroblasts migrating in response to damage, although not formally demonstrated yet, had been already suggested on the basis of immunohistochemistry and MRI analyses using transplanted cells. Indeed, a strong tropism of neural precursors for glioblastomas has been described in mice [22], [44]. In addition to the migration ability of exogenous precursors, some studies demonstrated it for endogenous neural precursors. For example, using a nestin-GFP transgenic, Glass et al. [44] have shown, in a murine experimental glioblastoma model, that nestin^+^ cells originating in the subventricular zone migrated towards the tumor vicinity within 4–14 days. These cells were ki67^+^, mushashi^+^, NG-2^+^, GFAP^+^, PSA-NCAM^+^ or DCX^+^, indicating the migration of committed and noncommitted precursors. The absence of unique cell surface markers for neural stem cells or for neuroblasts has impaired the *in vivo* study of particular cell phenotypes migration. Our data show that by combining the mAb Nilo2 with magnetic glyconanoparticles (Nilo2-mGNPs), the Nilo2 mAb *in vivo* zeroed in the complexes on neuroblasts, allowing the selective tracking of endogenous neuroblast progenitors *in vivo* by MRI. Indeed, Nilo2-mGNPs identified neuroblasts within their niches in the absence of additional damage. In addition, following injection of CT-2A cells, it also identified neuroblast cells surrounding the tumor, as demonstrated by MRI and confirmed by immunohistochemistry. The obtained MRI patterns were specific for Nilo2, since both anti-hp53 and anti-mouse CD3ε conjugated to the same mGNPs failed to show signals on neuroblast niches or surrounding the CT-2A tumor. In addition, confocal microscopy showed that the Nilo2-mGNP complexes remained intact *in vivo* for at least 23 days.

Complementary experiments in which, Nilo2-mGNPs were contralaterally injected after generating a tumor, or in which Nilo2-mGNPs were injected first (favoring binding of the mGNPs to Nilo2^+^ cells in their niches), both gave similar results, namely that Nilo2-mGNPs were detected surrounding the tumor cells after a short time-period. In addition, in grafts localized in more lateral positions, the time for the Nilo2-mGNP-labelled cells to reach the damage site increased. Interestingly, in experiments where binding of the Nilo2-mGNPs to neuroblasts in their niches was favored, MRI experiments demonstrated a continuous accumulation of Nilo2^+^ cells, starting as early as 5 h following injection of the CT-2A cells. Furthermore, Nilo2^+^ cells with migrating cell morphology (elongated nuclei and cytoplasmic projections) were detected in the *corpus callosum* at short times. Taken together, these data indicate that the accumulation of type I neuroblasts (Nilo2^+^) surrounding the tumor cells is due to an orderly migration of these cells from their brain niches (estimated speed range 150–700 µm/h) towards the lesion site, implying a fast damage response (less than 24 h), rather than to migration of brain-scattered neuroblasts or being the result of cell de-differentiation. The migration occurred either tangentially with a rostrocaudal pattern, throughout the entire SVZ, or in a radial pattern through the parenchyma at the contralateral hemisphere that crosses the high-dense structures of the *corpus callosum*.

In contrast to previous reports using *in situ* injection of a high concentration micron-sized iron oxide particles (MPIO) [17], and where only a small percentage of the migrating neuroblasts were labeled [15], [16], in this work, where a monoclonal antibody was coupled to the contrast agent, most of the resident neuroblasts were labeled without cellular damage and detected by MRI, even using a reduced amount of nanoparticles (0.11 µg Fe/mouse). It should be noted that the amount of Nilo2 used (0.20 µg Nilo2/mouse) is at least 60 times less than the minimal amount in which an effect on either neurosphere proliferation or differentiation *in vitro* could be detected [35]. These data support the notion that Nilo2 mAb zeroed in the mGNPs on neuroblast type I cells, which, as a consequence of a localized damage migrated labeled with Nilo2-mGNPs.

To exclude that some of the hypointense signals detected by MRI were background signals due to inflammation, iron accumulation due to hemorrhage or tumor growth, we included, in addition to controls with irrelevant antibodies coupled to the mGNPs (anti-hp53, anti-mouse CD3ε) or PBS injection, appropriate controls for each animal, namely the MRI data of the same animal prior to Nilo2-mGNP injection in the contralateral hemisphere (i.e. d+12 in Figure 3).

These data allowed us to envisage that neuroblast migration in response to a brain tumor might represent an attempt to repair the damaged tissue [42], [44]. In the CT-2A model used on this work, however, the fast tumor evolution (mice died within four weeks) hampered any possible benefit of the migrating neuroblasts.

Preliminary data on epilepsy models (G.E. unpublished results) suggest that neuroblast migration in response to damage is not restricted to this tumor model, since it also takes place following active peaks in some neurodegenerative diseases, opening up the possibility of using neuroblast migration for the early detection of brain lesions in neurodegenerative diseases where a link between damage with the etiopathology has not yet been fully established. In fact, this correlation could have strong implications in the prognosis and treatment of these diseases. Moreover, we have already demonstrated that other Nilo monoclonal antibodies had the ability to target nanoparticles to a particular cell subpopulation [45]. Fluorescent-magnetic glyconanoparticles conjugated to appropriate antibodies have been shown to specifically tag minor population of leucocytes (0.01%) in the whole human blood [37]. Thus, the use of nanoparticles is not necessarily restricted to the nervous system and could be used for locating minor populations such as stem cells in their niches, identification of cancer stem cells, or tracking migration of other cell types in the body, including metastatic cells.
